# Inference of differential kinase interaction networks with KINference

**DOI:** 10.1093/bioinformatics/btaf349

**Published:** 2025-06-20

**Authors:** Nicolai Meyerhöfer, Nevan J Krogan, Benjamin J Polacco, David B Blumenthal

**Affiliations:** Department Artificial Intelligence in Biomedical Engineering (AIBE), Friedrich-Alexander University Erlangen-Nürnberg (FAU), 91052 Erlangen, Germany; Quantitative Biosciences Institute (QBI), University of California, San Francisco, CA 94158, United States; Quantitative Biosciences Institute (QBI), University of California, San Francisco, CA 94158, United States; Department of Cellular and Molecular Pharmacology, University of California, San Francisco, CA 94158, United States; Gladstone Institute of Data Science and Biotechnology, J. David Gladstone Institutes, San Francisco, CA 94158, United States; Quantitative Biosciences Institute (QBI) COVID-19 Research Group (QCRG), University of California, San Francisco, CA 94158, United States; Quantitative Biosciences Institute (QBI), University of California, San Francisco, CA 94158, United States; Gladstone Institute of Data Science and Biotechnology, J. David Gladstone Institutes, San Francisco, CA 94158, United States; Quantitative Biosciences Institute (QBI) COVID-19 Research Group (QCRG), University of California, San Francisco, CA 94158, United States; Department Artificial Intelligence in Biomedical Engineering (AIBE), Friedrich-Alexander University Erlangen-Nürnberg (FAU), 91052 Erlangen, Germany

## Abstract

**Motivation:**

Differential kinase interaction networks (DKINs) are networks containing kinase–substrate links that are differentially active between two conditions. Existing methods are either able to predict condition-agnostic kinase–substrate links or condition-specific differential kinase activity, but do not provide differential kinase–substrate links. Moreover, existing methods for predicting kinase–substrate links usually rely on curated biochemical knowledge. Thus, there is a lack of data-driven DKIN inference methods that are also applicable when prior knowledge is scarce.

**Results:**

To address this need, we present KINference. KINference combines computation of a baseline KIN representing the space of all possible kinase–substrate links with filters applied to nodes and edges to identify differentially active subnetworks that are relevant in the context of a specific phosphoproteomics dataset. For the node filters, we rely on functional relevance and differential phosphorylation scores; for the edge filters, we make use of prize-collecting Steiner trees and correlations between phosphorylation sites of kinases and their target proteins. Tests on two phosphoproteomics datasets (kinase inhibition in breast cancer cells, SARS-CoV-2 infection in Calu-3 cells) show that the proposed filters produce significant results in terms of overlap with known interactions between kinases and phosphorylation sites. Furthermore, a case study on the SARS-CoV-2 infection data, suggests a potential host pathway linked to virus replication, showcasing the process of hypothesis generation utilizing DKINs computed by KINference.

**Availability and implementation:**

KINference is available as an R package at https://github.com/bionetslab/KINference and https://doi.org/10.5281/zenodo.15411150. Scripts to reproduce the results are available at https://github.com/bionetslab/KINference-Evaluation-Scripts and https://doi.org/10.5281/zenodo.15424599.

## 1 Introduction

Kinases play a major role in cellular signaling by mediating the phosphorylation of target proteins, a process critical for regulating a multitude of cellular functions such as metabolism, cell growth, cell division, cell differentiation and many more (Manning *et al.* 2002a,b). Protein phosphorylation is the addition of a phosphate group to serine (SER), threonine (THR), or tyrosine (TYR) residues—so-called phosphorylation sites or substrates. It is a reversible post-translational modification (PTM) that can alter the activity, localization, and interaction partners of a protein (Narayanan and Jacobson 2009, Deribe *et al.* 2010). This modification is catalyzed by kinases and reversed by phosphatases, creating a dynamic regulatory system that ensures cellular homeostasis and responsiveness to environmental cues. The human kinome consists of over 500 kinases that make up around 2% of the human proteome, and there are over 700 000 assumed phosphorylation sites in a typical eukaryotic cell (Ubersax and Ferrell 2007). Additionally, kinases regulate each other by phosphorylation, giving rise to a complex network of kinase–kinase interactions. Changes to this system can affect key cellular processes and are major drivers for diseases (Pang *et al.* 2022).

A plethora of biochemical knowledge on kinase signaling pathways has been accumulated and stored in public databases such as KEGG (Kanehisa *et al.* 2023), Reactome (Milacic *et al.* 2024), or OmniPath (Türei *et al.* 2021). Even dedicated databases like PhosphoSitePlus (Hornbeck *et al.* 2015) for kinase–protein relationships have been developed. However, these databases often suffer from a study bias toward a small set of highly studied proteins that are prominent in major diseases (Gillis *et al.* 2014). Therefore, data-driven approaches are necessary to unravel the interactions of less-studied kinases.

Several tools to chart the space of less studied kinase–substrate links exist. Among them are methods from Invergo *et al.* (2020), who developed a supervised machine learning model that estimates the probability of regulatory relationships between human kinases utilizing tree-based regression models. Phosformer (Zhou *et al.* 2023) is an explainable protein language model that predicts the probability of phosphorylation given the sequence of a kinase and the peptide sequence surrounding a phosphorylation site. KinomeXplorer (Horn *et al.* 2014) is a naïve Bayes method for scoring proximity scores based on STRING (Szklarczyk *et al.* 2023) and proximity scores based on NetPhorest (Miller *et al.* 2008). However, the predicted kinase–substrate links and resulting kinase interaction networks (KINs) of the respective tools are still context-agnostic in the sense that they cannot be tailored to specific phosphoproteomics datasets provided by the users. Such data-driven approaches are becoming increasingly feasible as the field of high-throughput phosphoproteomics advances with technologies such as liquid-chromatography tandem mass spectrometry (LC-MS/MS) (Paulo and Schweppe 2021).

On the experimental side, Buljan *et al.* (2020) performed a large-scale affinity purification-mass spectrometry (AP-MS) experiment for more than 300 kinase baits of all kinase families and used it to *de novo* construct a KIN that does not inherit the study bias of large-scale databases. However, AP-MS is largely used for detecting stable interactions, whereas phosphorylation events are fast and unstable (Blazek *et al.* 2015). Therefore, this approach cannot capture phosphorylation-mediated kinase interactions. Moreover, the obtained KIN is again context-agnostic and does not capture the differentially active kinase–substrate links, i.e. does not return a differential KIN (DKIN).

On the other hand, there are statistical methods that can predict differential kinase activity, such as KSTAR (Crowl *et al.* 2022) or the method by Johnson *et al.* (2023). However, these tools only give a score for the activity of a kinase in a given dataset, condition, or sample and do not return condition-specific kinase–substrate links, again, they again do not return a DKIN.

To address these limitations, we developed KINference. KINference provides inbuilt support to construct a baseline KIN, relying on position-specific-socring-matrices (PSSMs) scores from two recent large-scale experiments by Johnson *et al.* (2023) and Yaron-Barir *et al.* (2024). PSSMs are explained in detail in Section 1.1, available as supplementary data at *Bioinformatics* online. This baseline KIN serves to chart the space of all possible kinase–substrate interactions that may be relevant for the underlying biological mechanism. Alternatively, any other baseline KIN can be used. The baseline KIN is then filtered for functional relevance of phosphorylation sites or dataset-specific differential phosphorylation intensity. These two node filters are supplemented with two edge filters. The first edge filter is based on dataset-specific correlation of chains of phosphorylation events and can be used when sample sizes are large enough to support robust computation of correlation coefficients. The second edge filter models the task of finding a differential subnetwork as an instance of the prize-collecting Steiner tree (PCST) problem and can also be used when only data for very few samples is available. Thus, KINference returns a data-driven DKIN that highlights the condition-specific kinase–substrate interactions.

We tested KINference on data from two large-scale phosphoproteomic experiments (Wilkes *et al.* 2015, Bouhaddou *et al.* 2023), showing that all filtering steps available in KINference significantly outperform random baselines in terms of retrieval of known kinase interactions annotated in OmniPath. Moreover, a closer analysis of the results obtained for the SARS-CoV-2 infection data by Bouhaddou *et al.* (2023) showcases how DKINs computed by KINference can be used for hypothesis generation and identification of host pathways involved in viral infections.

## 2 Materials and methods

### 2.1 Overview of KINference

**Figure 1.**
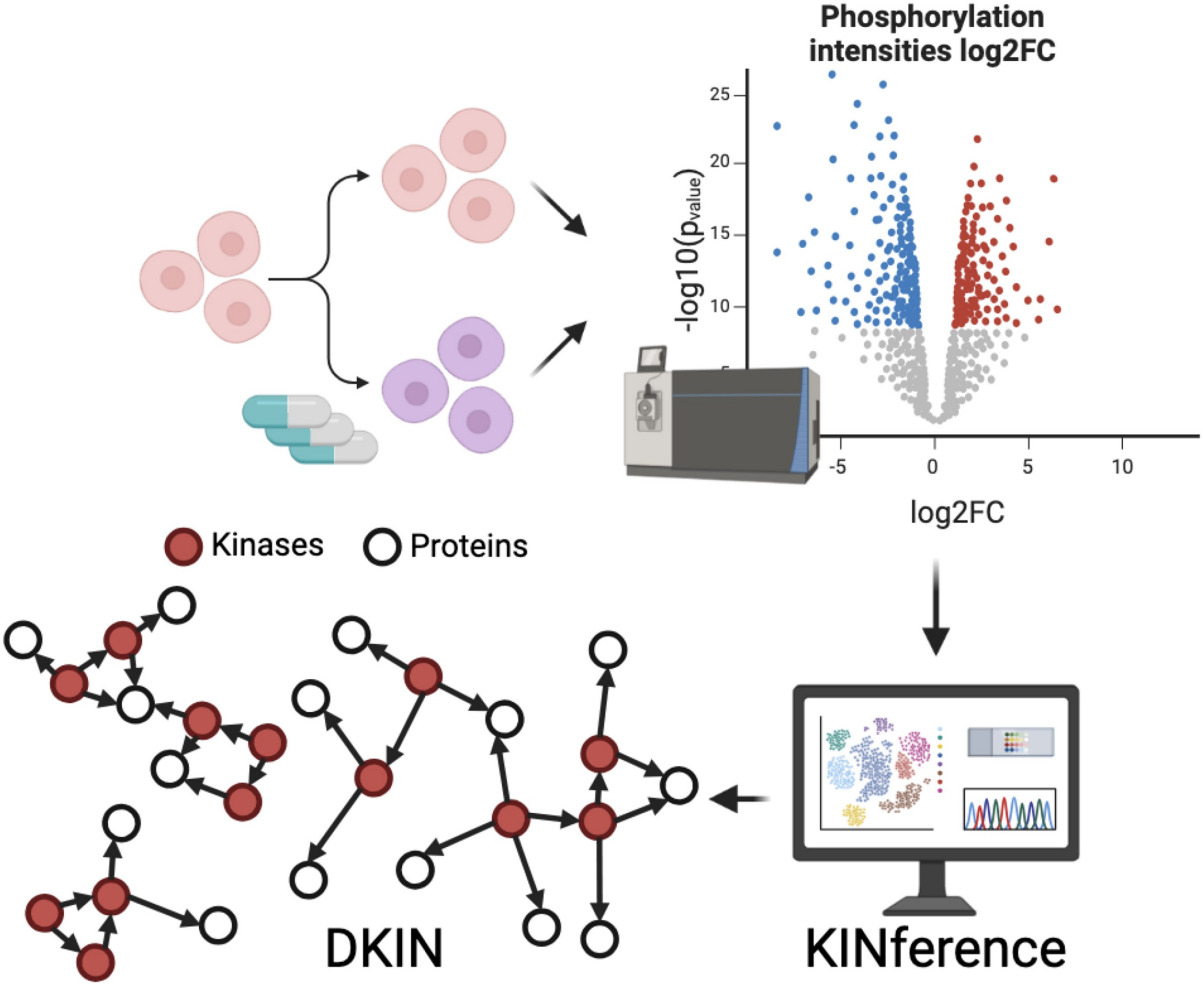
Overview of KINference’s concept. KINference expects log2-fold changes of phosphorylation site intensities of an MS experiment and outputs a DKIN containing the differentially active kinase–kinase and kinase–protein interactions.

KINference addresses the task of elucidating biological mechanisms that drive diseases by inferring a condition-specific DKIN from phosphoproteomics data (Fig. 1). Toward this end, the problem of computing a DKIN is decomposed in two steps. First, KINference computes a condition-agnostic baseline KIN that charts the space of possible kinase–substrate interactions. Then, the baseline KIN is mined for a differentially active subnetwork containing the relevant differentially active kinase–substrate links for *de novo* hypothesis generation.

### 2.2 Input and output of KINference

As input, KINference requires matrices $X^{1}=(x_{i,s}^{1})\in R^{D_{1}\times S}$ and $X^{0}=(x_{i,s}^{0})\in R^{D_{0}\times S}$ of phosphorylation intensity measurements, where $D_{1}$ and $D_{0}$ are sets of samples from, respectively, a target and a control condition. $S=S^{\text{SER}/\text{THR}}\cup S^{\text{TYR}}$ is the set of all serine/threonine and tyrosine phosphorylation sites measured for both conditions. If no raw phosphorylation intensities are available, the user can alternatively provide a vector $f=(f_{s})\in R^{S}$ that contains the log2-transformed fold change in phosphorylation intensity between the two conditions for a set of phosphorylation sites *S*. Phosphorylation sites s∈S are expected to be provided as UniProt IDs (UniProt Consortium 2023), which annotate the phosphorylation sites with the ID of the corresponding proteins and the sequence positions of the phosphorylated residues.

**Figure 2.**
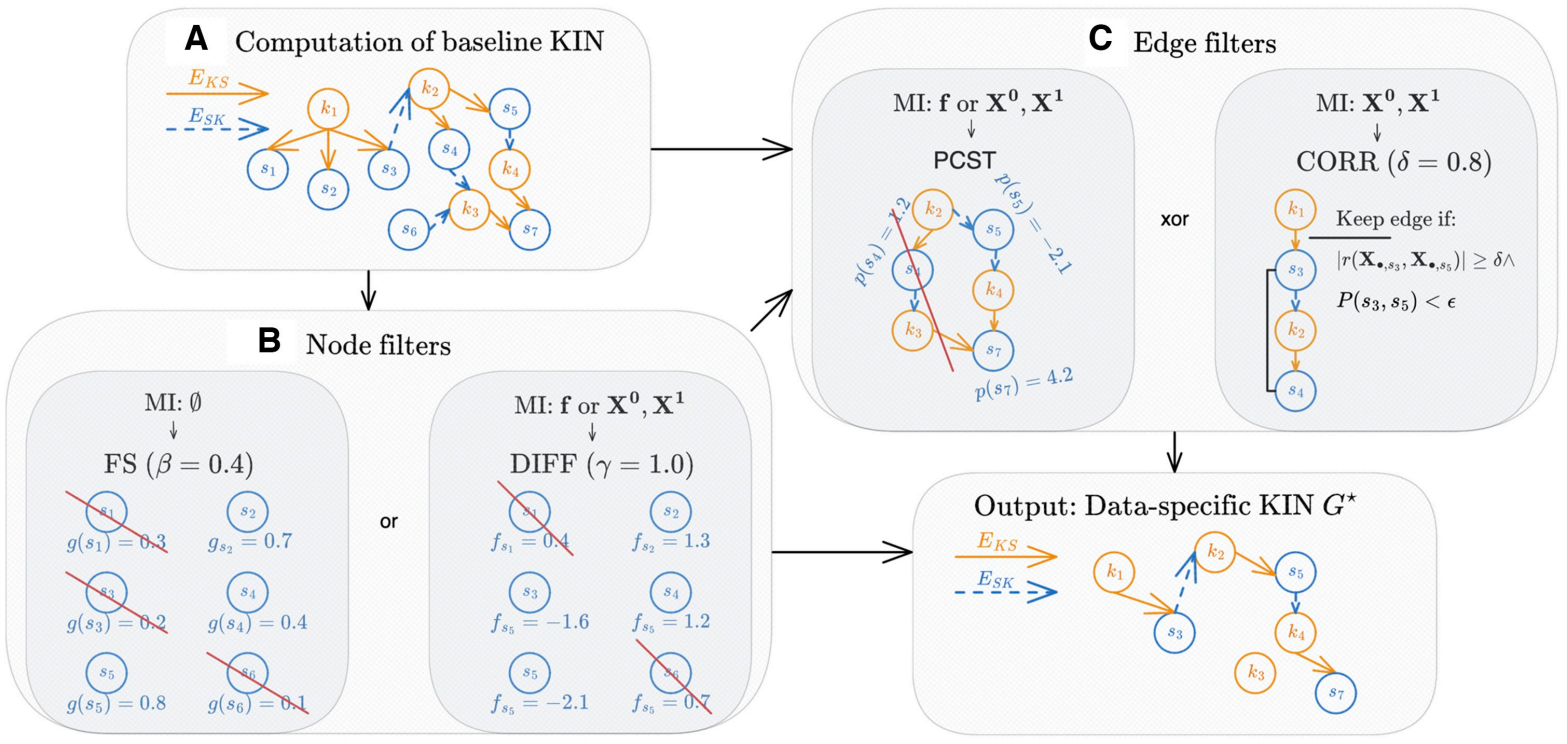
Overview of KINference together with minimal input (MI) required for the individual steps. (A) First, we construct a baseline KIN comprised of kinase–substrate edges obtained based on the PSSM scoring devised by Johnson *et al.* (2023) and substrate-kinase edges that link phosphorylation sites on a kinase to this kinase. (B) Node filters based on, respectively, functional relevance of phosphorylation sites and dataset-specific differential phosphorylation may be applied individually or combined. (C) Edge filters relying on co-phosphorylation or subnetwork mining via PCSTs.

Output of KINference is a directed bipartite network


(1) $$G^{⋆}=(\underset{\text{nodes}}{\underset{︸}{K^{⋆}\cup S^{⋆}}},\underset{\text{edges}}{\underset{︸}{E_{KS}^{⋆}\cup E_{SK}^{⋆}}}),$$

where $K^{⋆}\subset K$ is a subset of the set of all kinases $K=K^{\text{SER}/\text{THR}}\cup K^{\text{TYR}}$ for which PSSM scores are provided by Johnson *et al.* (2023) and Yaron-Barir *et al.* (2024) or are contained in an alternatively provided baseline KIN. Kinases $K\setminus K^{⋆}$ are removed during the filtering steps if a kinase $k\in K\setminus K^{⋆}$ has no more outgoing interactions $e\in E_{KS}^{⋆}$ or incoming edges to one of its phosphorylation sites left, as singletons do not provide any information in the interaction network. $S^{⋆}\subset S$ is a subset of the set of measured phosphorylation sites *S*. $E_{KS}^{⋆}\subseteq K^{⋆}\times S^{⋆}$ contains directed edges from a kinase *k* to a substrate *s* phosphorylated by it. $E_{SK}^{⋆}\subseteq S^{⋆}\times K^{⋆}$ contains directed edges from phosphorylation sites *s* of a kinase *k* to this kinase. Figure 2 provides an overview of how KINference infers $G^{⋆}$ from the input $X^{1}$, $X^{0}$, and/or f. The individual steps are detailed below.

### 2.3 Construction of baseline KIN

The baseline KIN $G=(K\cup S,E_{KS}\cup E_{SK})⊇G^{⋆}$ is a supergraph of the final network $G^{⋆}$. To compute *G*, we rely on data by Johnson *et al.* (2023) and Yaron-Barir *et al.* (2024), each of whom conducted a large-scale positional scanning peptide array (PSPA) analysis. Johnson *et al.* (2023) curated PSSMs for $|K^{\text{SER}/\text{THR}}|=303$ human SER and THR kinases at 9 positions surrounding SER or THR phosphorylation sites. Yaron-Barir *et al.* (2024) collected PSSMs for $|K^{\text{TYR}}|=93$ human TYR kinases at 10 positions surrounding TYR phosphorylation sites. Based on these PSSMs, Johnson *et al.* (2023) suggested a scoring mechanism that computes PSSM scores $r_{k,s^{\prime }}^{\text{SER}/\text{THR}}$ for every kinase $k\in K^{\text{SER}/\text{THR}}$ and every SER or THR phosphorylated substrate $s^{\prime }\in S^{\prime \;\text{SER}/\text{THR}}$, where $S^{\prime \;\text{SER}/\text{THR}}$ is a set of 82 756 high-confidence SER or THR phosphorylation sites curated by Ochoa *et al.* (2020). The same scoring mechanism is utilized by Yaron-Barir *et al.* (2024) to obtain PSSM scores $r_{k,s^{\prime }}^{\text{TYR}}$ for all kinases $k\in K^{\text{TYR}}$ and every TYR phosphorylated substrate $s^{\prime }\in S^{\prime \;\text{TYR}}$, which contains 5431 high-confidence TYR phosphorylation sites that were also collected in the experiment of Ochoa *et al.* (2020).

The PSSM scoring is done separately for the two types t∈{SER/THR,TYR} of kinases. For each phosphorylation site $s\in S^{t}\subseteq S$, we compute the PSSM scores $r_{k,s}^{t}$ for all $k\in K^{t}$ and the percentiles $p_{k,s}^{t}$ of these scores w. r. t. the background distribution $\left\{r_{k,s^{\prime }}^{t}|s^{\prime }\in S^{\prime t}\right\}$. We then sort the kinases $K^{t}$ in non-increasing order $k_{1},k_{2},\ldots ,k_{\left|K^{t}\right|}$ with respect to $p_{k_{i},s}^{t}$. A detailed explanation is given in Section 1.1, available as supplementary data at *Bioinformatics* online. Subsequently, we construct the set $K_{s}^{t}=\{k_{i}|1\leq i\leq n\wedge p_{k_{i},s}^{t}\geq \alpha \}$ of top *n* kinases with percentile at least α and define the overall set of kinase–substrate edges for one type *t* of kinases in the baseline KIN as the union


(2) $$E_{KS}^{t}=\underset{s\in S^{t}}{\cup }\{(k,s)|k\in K_{s}^{t}\}$$

of edges from kinases in $K_{s}^{t}$ to *s* over all substrates $s\in S^{t}$. Following the recommendations of Johnson *et al.* (2023), we use hyperparameters n=15 and α=0.9 for this step. The set of substrate-kinase edges $E_{SK}^{t}$ is simply defined as the set of all substrate-kinase pairs $(s,k)\in S^{t}\times K^{t}$ such that *s* is a phosphorylation site of the kinase *k*. Finally, the total kinase–substrate interactions $E_{KS}=E_{KS}^{\text{SER}/\text{THR}}\cup E_{KS}^{\text{TYR}}$ and substrate-kinase edges $E_{SK}=E_{SK}^{\text{SER}/\text{THR}}\cup E_{SK}^{\text{TYR}}$ of the baseline KIN each consist of the union over both types *t* of kinases.

As a phosphoproteomics dataset may contain around 30 000–40 000 phosphorylation sites, the baseline KIN *G* will contain a large number of edges. To facilitate hypothesis generation, KINference therefore supports filtering steps to obtain subgraphs of *G* that are relevant in the context of the user-provided input data. The final DKIN $G^{⋆}=(K^{⋆}\cup S^{⋆},E_{KS}^{⋆}\cup E_{SK}^{⋆})$ is then the result of applying all filtering steps selected by the user.

### 2.4 Node filtering techniques

Here, we present two node filtering methods based on a functional score (FS) and a differential score (DIFF) for selecting significant parts of the inferred baseline KIN. These can be applied individually, combined, or not at all (Fig. 2B). Both filters select a subset $S^{\text{FILTER}}\subseteq S$ of the substrates contained in the baseline KIN. The filtered KIN is then computed as the induced subgraph $G[K\cup S^{\text{FILTER}}]$ (i.e. no kinases are filtered out).

For the FS filter, we use the machine learning model developed by Ochoa *et al.* (2020). Using evolutionary, regulatory, and structural information, the model assigns a functional score g(s) to each phosphorylation site, which estimates the relevance of *s* for the fitness of the organism. In KINference, we use the trained model provided by Ochoa *et al.* (2020) (trained on 2638 annotated phosphorylation sites) to filter the substrate nodes of the KIN as


(3) $$S^{\text{FS}}=\{s\in S|g(s)\geq \beta \},$$

where *S* is the set of substrates in the baseline KIN (potentially already filtered via the DIFF filter detailed below) and β∈[0,1] is a hyperparameter which we set to β=0.4 by default, following the recommendation by Ochoa *et al.* (2020). The FS filter helps remove phosphorylation sites that are less probable to have an impact on the affected protein. However, a major constraint of the FS filter is that it was only designed for human phosphorylation sites. Therefore, phosphorylation sites of other species have to be either mapped to the corresponding human phosphorylation sites or this filter cannot be used. KINference only supports mapping of phosphorylation sites from mice to the respective human phosphorylation sites. Phosphorylation sites of other species have to be mapped by the user in advance. Just like the construction of the baseline KIN, the FS filter is still independent of the phosphoproteomics data provided by the user, but it suffers from being literature-based and, therefore, potentially introduces literature bias back into the otherwise data-driven method. This may lead to the removal of relevant phosphorylation sites in less-studied conditions.

KINference’s DIFF filter serves to obtain a DKIN that only contains interactions involving phosphorylation sites whose phosphorylation intensities differ between two conditions. We define the filter as


(4) $$S^{\text{DIFF}}=\{s\in S||f_{s}|\geq \gamma \},$$

where *S* is the set of substrates in the baseline KIN (potentially already filtered via the FS filter) and $\gamma \in R^{+}$ is a hyperparameter (default: γ=1). If the vector $f=(f_{s})$ of log2-transformed fold changes in phosphorylation intensity is provided as input by the user, KINference uses this data for the DIFF filter. If the user instead provides matrices $X^{1}=(x_{i,s}^{1})\in R^{D_{1}\times S}$ and $X^{0}=(x_{i,s}^{0})\in R^{D_{0}\times S}$ of phosphorylation intensities for two conditions, we instead compute f as


(5) $$f_{s}=\underset{i\in \{1,\ldots ,|D_{0}|\}}{\text{mean}}{\;\text{log}\;}_{2}\left(x_{i,s}^{1}/x_{i,s}^{0}\right),$$

if $X^{1}$ and $X^{0}$ contain data for paired samples, where the same underlying biological replicate is used before, e.g. administering a drug to a subset of the replicate, and as


(6) $$f_{s}={\;\text{log}}_{2}\left(\underset{i\in \{1,\ldots ,|D_{1}|\}}{\text{mean}}x_{i,s}^{1}/\underset{i\in \{1,\ldots ,|D_{0}|\}}{\text{mean}}x_{i,s}^{0}\right),$$

otherwise.

### 2.5 Edge filtering techniques

We introduce two methods to filter the edges of the KIN. The first edge filter is based on correlation scoring (CORR) and requires that a matrix $X\in R^{D\times S}$ of phosphorylation intensities is available for at least one condition with sufficiently many samples to compute robust correlation coefficients. The second edge filter utilizes prize-collecting Steiner trees (PCST) and only requires the availability of log2-fold changes f (which can be computed also for small samples sizes as long as data for two conditions is provided). The edge filters can be optionally combined with the aforementioned node filtering approaches as shown in Fig. 2C. We consider them as edge filters because the CORR and PCST filter only remove individual edges. A node is deleted if all its adjacent edges are filtered out because singletons do not provide useful information for an interaction network.

Correlation analysis between phosphorylation sites has been already used to detect co-phosphorylation patterns of individual kinases (Ayati *et al.* 2019). However, differently from the work of Ayati *et al.* (2019), the CORR filter aims to detect effects of phosphorylation of a substrate of a kinase on its activity. The CORR filter acts on kinase–substrate edges $(k,s)\in E_{KS}$ where *s* is a phosphorylation site on another kinase k(s) (we use the notation $E_{KS^{\prime }}\subset E_{KS}$ to the denote the set of these edges). It removes the edge $(k,s)\in E_{KS^{\prime }}$ if phosphorylation of *s* has no effect on the activity of k(s). All interactions between kinases and proteins that are not kinases are kept in the network. To assess the edges $E_{KS^{\prime }}$, we compute Pearson correlation coefficients $r(X_{●,s},X_{●,s^{\prime }})$ and corresponding adjusted *P*-values $P(s,s^{\prime })$ for all substrates $s^{\prime }\in N_{+}(k(s))$ targeted by k(s). We provide the choice between Benjamini–Hochberg correction, Bonferroni correction, and no multiple testing correction for adjusting the *P*-values. $N_{+}(k(s))$ is the set of all out-neighbors of k(s) in the current (possibly already filtered) KIN and $X_{●,s}$ and $X_{●,s^{\prime }}$ are the columns of *s* and $s^{\prime }$ in the phosphorylation intensity matrix X. A positive correlation $r(X_{●,s},X_{●,s^{\prime }})$ indicates that the phosphorylation of site *s* on kinase k(s) leads to a gain of function in its ability to phosphorylate $s^{\prime }$. The opposite holds for the case of negative correlation. If no correlation exists, the phosphorylation of *s* has no impact on k(s)’s ability to phosphorylate $s^{\prime }$.

With these preparations, we define the CORR filter as $E_{KS}^{\text{CORR}}=(E_{KS}\setminus E_{KS^{\prime }})\cup E_{KS^{\prime }}^{\text{CORR}}$, where


(7) $$\begin{array}{c} E_{KS^{\prime }}^{\text{CORR}}=\{(k,s)\in E_{KS^{\prime }}|\exists s^{\prime }\in N_{+}(k(s)):\\ |r(X_{●,s},X_{●,s^{\prime }})|\geq \delta \wedge P(s,s^{\prime })<\epsilon \}, \end{array}$$

and the minimum effect size and the significance cutoff are set to δ=0.8 and ϵ=0.05 by default. The CORR filter hence keeps an edge $(k,s)\in E_{KS^{\prime }}$ if there is at least one substrate $s^{\prime }$ targeted by k(s) such that phosphorylation of *s* is correlated with phosphorylation of $s^{\prime }$. If data matrices $X^{1}$ and $X^{0}$ are provided for two conditions, KINference applies the CORR filter individually and returns one filtered DKIN per condition.

MS studies often conduct experiments with few replicates (often just three or four) because of time and financial reasons. In such settings, the CORR filter cannot be used. Therefore, we propose another edge filter based on the PCST problem (Karp 1972) that can be used also when sample sizes are very small. The PCST problem is defined on an undirected graph $H=(V_{H},E_{H})$ with positive edge weights $w:E_{H}\to R^{+}$ and node prizes $p:V_{H}\to R^{+}$ and returns a subtree $T=(V_{T},E_{T})$ of *H* (i.e. $V_{T}\subseteq V_{H}$ and $E_{T}\subseteq E_{H}$) that minimizes $\sum_{e\in E_{T}}w(e)+\sum_{v\in V_{H}\setminus V_{T}}p(v)$. That is, the problem asks to collect nodes $v\in V_{H}$ with high prizes p(v) while minimizing the edge costs w(e) of the edges $e\in E_{H}$ used to connect the collected nodes.

The PCST problem has been successfully used as an underlying mathematical model in disease module mining methods that aim at extracting subgraphs of protein-protein interaction networks which are enriched with disease-associated proteins (Levi *et al.* 2021, Bernett *et al.* 2022, Sarkar *et al.* 2023). In KINference, we follow a similar approach and use the PCST problem to model the task of extracting subgraphs enriched with differentially phosphorylated substrates from the baseline (or already pre-filtered) KIN. To do so, we first remove the directionality of the edges in the KIN by replacing both substrate-kinase edges (s,k) and kinase–substrate edges (k,s) with undirected edges *ks*. Then, we define node prizes as


(8) $$p(v)=\{\begin{array}{cc} \left|f_{s}\right| & \text{if}\;v=s\in S\;\text{is}\;a\;\text{substrate}\;\text{node}\\ \gamma & \text{if}\;v=k\in K\;\text{is}\;a\;\text{kinase}\;\text{node} \end{array},$$

where $f_{s}$ is the log2-fold change in phosphorylation intensity for substrate *s* (provided by the user or computed from $X^{1}$ and $X^{0}$ as detailed in Equations (5) and (6)). The node prizes of all kinases in *K* are set to γ, the user-set parameter for the DIFF filter. We do this to incentivize the PCST model to collect kinase nodes with the same priority as minimally differentially phosphorylatated substrates. The edge weights *w* are uniformly set to 1 such that the PCST model optimizes for subnetworks with the smallest possible number of edges, facilitating hypothesis generation.

The PCST problem is NP-hard but well-performing approximate solvers exist. In KINference, we use Chinmay *et al.* (2014)’s implementation of the dual approximation algorithm by Goemans and Williamson (1995), which has an approximation guarantee of 2. The algorithm computes a subtree $T=(V_{T},E_{T})$ of the undirected version of the baseline or pre-filtered KIN $G=(V,E_{KS}\cup E_{SK})$, and KINference’s PCST filter


(9) $$\begin{array}{c} V^{\text{PCST}}=V^{T}\\ E^{\text{PCST}}=\{(k,s)\in E_{KS}|ks\in E_{T}\}\cup \{(s,k)\in E_{SK}|ks\in E_{T}\} \end{array}$$

returns this tree with directionality of the edges restored.

### 2.6 Test datasets

We used large-scale LC-MS/MS datasets by Wilkes *et al.* (2015) and Bouhaddou *et al.* (2023). Wilkes *et al.* (2015) measured the effect of different drugs that inhibit phosphoinositide-3-kinase (PI3K) in breast cancer cell lines that are resistant to the respective drugs compared to a control condition across 6 replicates for each condition (three technical and two biological). The data is provided as log2-transformed fold changes of 15 327 phosphorylation intensities between the drug and the control condition. Of those, 7555 log2-fold changes reach the 0.05 significance threshold [*P*-values obtained from the original publication by Wilkes *et al.* (2015)] and 3966 sites are shared with Ochoa *et al.* (2020). For our work, we used the data for PI3K inhibitor pictilisib. Bouhaddou *et al.* (2023) performed a SARS-CoV-2 variant analysis. For this, they infected Calu-3 cells with one of the SARS-CoV-2 lineages, or a no-virus mock infection as a control, and then measured phosphorylation intensities after 10 and 24 h post infection. Nine samples were curated for every condition and time point and the data are provided as matrices of phosphorylation intensity measurements. We used the data for the Beta variant [46 541 phosphorylation intensity measurements, 23 719 sites shared with Ochoa *et al.* (2020)] and the control cells [35 649 phosphorylation intensity measurements, 25 327 shared with Ochoa *et al.* (2020)] at time point 10 h.

### 2.7 Evaluation strategy and baselines

To validate KINference, we computed overlaps $\left|E_{KS}^{⋆}\cap E_{KS}^{\text{OmniPath}}\right|$ between the sets $E_{KS}^{⋆}$ of kinase–substrate edges contained in the DKINs returned by KINference when run with different node and edge filters and the set $E_{KS}^{\text{OmniPath}}$ of known kinase–substrate interactions annotated in OmniPath. We chose OmniPath because it provides broad coverage by combining eleven PTM databases containing 39 201 enzyme-PTM relationships (1821 enzymes and 16 467 PTM sites) with 94% of these interactions being phosphorylations. In the absence of existing methods for data-driven DKIN inference, we compared the DKINs computed by KINference to two randomized baselines. For the first baseline, we randomly selected $\left|E_{KS}^{⋆}\right|$ edges from the set $E_{KS}$ of kinase–substrate edges of the baseline KIN and then computed overlaps with $E_{KS}^{\text{OmniPath}}$. We repeated this 1000 times, leading to a distribution of 1000 overlap scores obtained by random selections of edges from the baseline KIN. For the second baseline, we randomly rewired the edges $E_{KS}^{⋆}$ of the DKIN while preserving the node degrees, using the rewire() and keeping_degseq() functions of the R igraph package (Csardi and Nepusz 2006). These functions ensure that the randomly rewired edges can only be physiologically relevant edges by maintaining the directionality from kinases to substrates during the randomization process. Then, we computed overlaps between the rewired kinase–substrate edges and $E_{KS}^{\text{OmniPath}}$. Again, we repeated this 1000 times, and thus obtained 1000 overlap scores that represent the expected OmniPath overlap of DKINs that respects the node connectivity of the DKIN. We then used the one-sided *Z*-test to assess if KINference’s OmniPath overlaps are larger than the overlap distributions obtained for the two baselines. We carried out a hyperparameter evaluation for the baseline KIN-specific hyperparameters α and *n*, and the filter-specific hyper-parameters β, γ, and δ utilizing the randomized filtering and randomized rewiring test. We computed the mean *P*-values and the effect size w.r.t. the number of edges remaining after filtering.

Furthermore, we assessed the literature bias of the different baseline KINs and DKINs by computing the PubMed coverage of the target proteins contained in the different networks. We define the PubMed coverage of a protein as the number of PubMed IDs indexed to this protein, relying on data from https://ftp.ncbi.nih.gov/gene/DATA/gene2pubmed.gz (downloaded on February 27, 2025). The larger the PubMed coverages, the stronger the literature bias of the networks.

We also compared KINference’s baseline KIN against the two condition-agnostic KINs: A KIN inferred by the machine learning model of Invergo *et al.* (2020) and the experimental AP-MS-based KIN by Buljan *et al.* (2020). To evaluate the capabilities of the baseline KIN to recover the edges in these KINs when run on a dataset containing phosphorylation intensities of the involved phosphorylation sites, we constructed two mock datasets consisting of all possible phosphorylation sites of the high-confidence targeted proteins contained in the KINs by Invergo *et al.* (2020) and Buljan *et al.* (2020), respectively. Moreover, on the dataset by Bouhaddou *et al.* (2023), we compared the baseline KIN to a KIN inferred by the state-of-the-art network reconstruction tool GRNBoost2 (Moerman *et al.* 2019), a gradient boosting method is typically used to infer gene regulatory networks (GRNs) from gene expression data. Running GRNBoost2 on the data by Wilkes *et al.* (2015) was not possible because of too small sample sizes. Further details on the comparisons of KINference’s baseline KIN against the two condition-agnostic KINs and the KIN inferred by GRNBoost2 are provided in Section 1.2, available as supplementary data at *Bioinformatics* online.

To evaluate the potential of KINference to detect dataset-specific phorsphorylation dynamics, we performed pathway enrichment analysis with g: Profiler (Kolberg *et al.* 2023), using KEGG (Kanehisa *et al.* 2023) as reference. As query sets, we used the sets of all genes corresponding to kinases and substrates contained in dataset-specific DKINs computed by KINference.

## 3 Results

This section is organized as follows: first, we analyze the effects of the hyperparameters of the baseline KIN and the downstream filters (Section 3.1). The best-performing hyperparameter combination is then fixed and used for comparing KINference’s baseline KIN to existing condition-agnostic KINs and a KIN inferred by GRNBoost2 (Section 3.2) and for a detailed analysis of the DKINs produced by various filter combinations (Section 3.3). All of these analyses rely on comparisons with OmniPath. Finally, Sections 3.4 and 3.5 provide qualitative analyses of DKINs obtained with the best-performing filter combinations.

### 3.1 Effect of hyperparameters controlling baseline KIN and filter behavior

**Figure 3.**
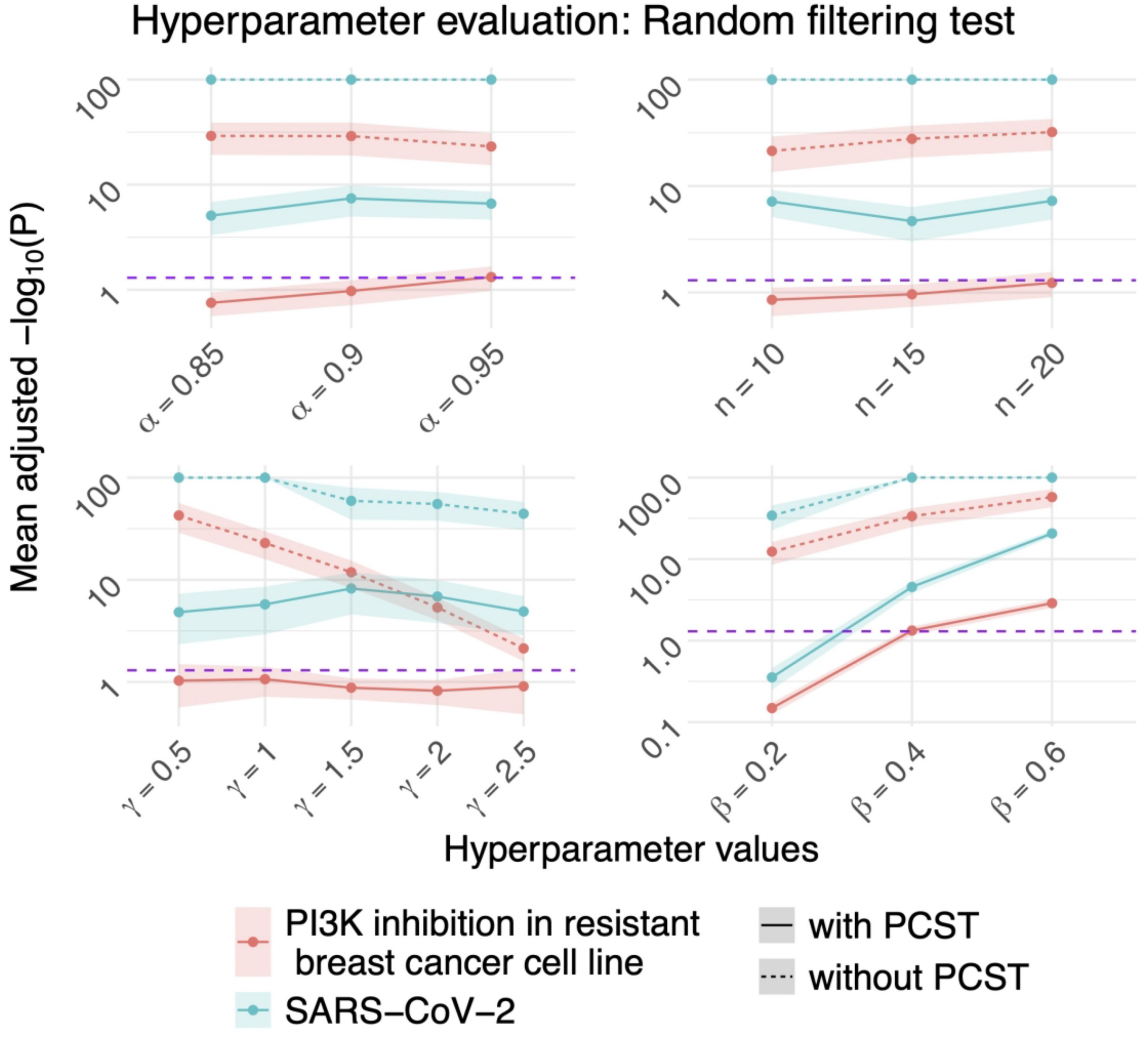
Hyperparameter evaluation of the randomized filtering test of the hyperparameters α, *n*, β, and γ for the two test datasets utilizing DIFF + FS (dashed lines) and PCST + DIFF + FS (solid lines) and the effect sizes. Mean adjusted $-{\;\text{log}\;}_{10}$-transformed *P*-values with the respective 5% confidence intervals are shown (one-sided *Z*-test; alternative hypothesis: OmniPath overlaps obtained with KINference’s filter are significantly larger than expected by chance; *P*-values are adjusted for multiple testing via Benjamini–Hochberg correction to account for the number of tested hyperparameter values). Purple lines: 0.05 significance threshold.

The random rewiring test returns significant mean *P*-values for all hyperparameter settings (Fig. 1, available as supplementary data at *Bioinformatics* online). This indicates that the baseline KIN obtained via the PSSM scoring already captures significant structures in the test datasets, which are also not lost by the downstream filtering. Furthermore, the baseline KIN specific hyperparameters α and *n* have no clear effect on the mean *P*-values after applying the DIFF, FS or PCST filter (Fig. 3). We chose α=0.9 and n=15 for all downstream analyses because these values are also used by the developers of the PSSM scoring (Johnson *et al.* 2023). Moreover, Fig. 3 shows that the FS filter significantly improves the mean *P*-values with increasing values of β and that the DIFF filter achieves significant results for 1.0≤γ≤2.0.

The PCST filter achieves significant results when run with β≥0.4. However, overall, the PCST filter does not perform well without prior node filtering. Consequently, it should be used in combination with the DIFF or FS filters, as these combinations produce the smallest and thus most interpretable networks (Fig. 2, available as supplementary data at *Bioinformatics* online).

The most significant literature bias is observed for the FS filter (Fig. 3, available as supplementary data at *Bioinformatics* online): Across both test datasets and both with and without additional PCST filtering, the hyperparameter β that controls the strength of the FS filter is significantly positively correlated with the PubMed coverages of the target proteins in the resulting DKINs (Table 1, available as supplementary data at *Bioinformatics* online). For the γ and δ parameters controlling the DIFF and CORR filters, we mostly observe no significant correlation with the PubMed coverages. In order to obtain small networks that are not highly prone to literature bias and also produce significant results, we use the DIFF + FS + PCST filter combination with γ=1.0 and β=0.4 for all downstream analyses.

**Figure 4.**
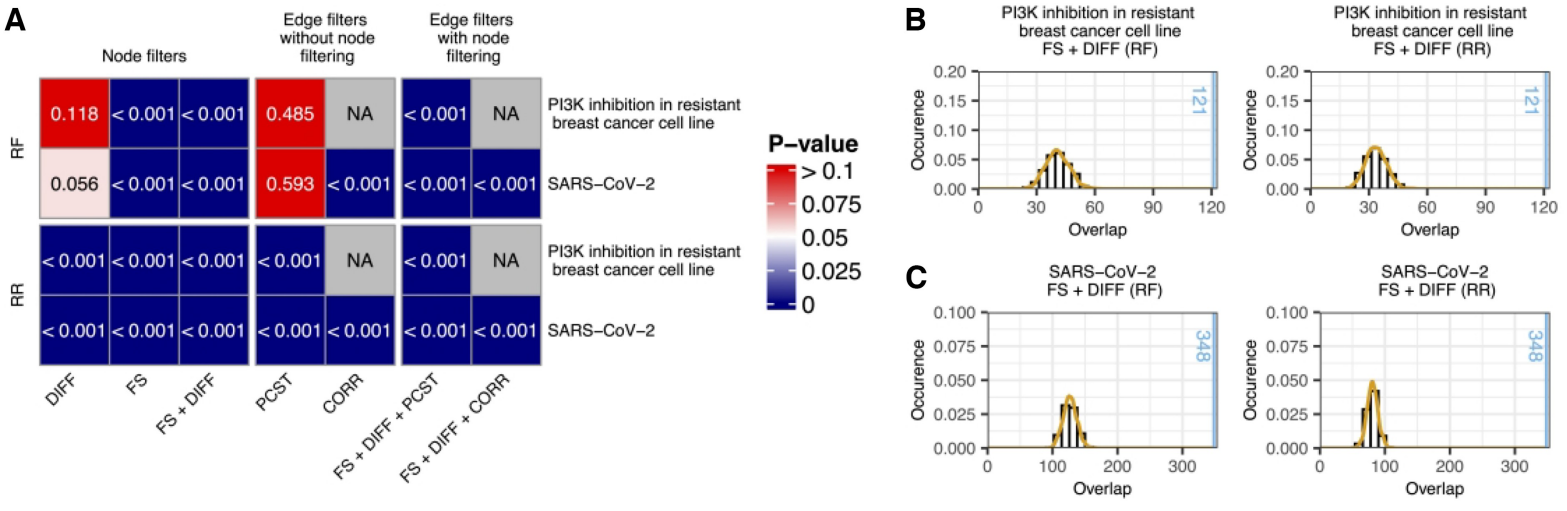
Results of OmniPath-based evaluation of DKINs obtained with different filter combinations. (A) *P*-values (one-sided *Z*-test) obtained when comparing OmniPath overlaps achieved with KINference and different combinations of node and edge filters against overlaps achieved via random filtering (RF) of the baseline KIN and randomized rewiring (RR) of the DKINs computed by KINference. *P*-values for all filter combinations are shown in Fig. 7, available as supplementary data at *Bioinformatics* online. (B, C) Distributions of OmniPath overlaps underlying the *P*-values of the FS + DIFF node filter combination shown in (A). Histograms and density plots visualize the overlap distributions obtained with the randomized baselines; the vertical blue lines show the overlaps of the DKINs computed with KINference. See Figs 8–12, available as supplementary data at *Bioinformatics* online for overlap distributions underlying all *P*-values.

Different values of δ have almost no effect on the behavior of the CORR filter: if an edge is found to be significant, it almost always has a high correlation (δ≥0.9) due to the low sample size. Furthermore, the CORR filter does not filter many edges because it can only remove kinase–kinase links, and the number of such links is much smaller than the number of kinase–protein links (Fig. 4, available as supplementary data at *Bioinformatics* online).

### 3.2 KINference’s baseline KIN compared to condition-agnostic KINs and a KIN inferred by GRNBoost2

KINference’s baseline KINs recover 88.1% of the 3701 interactions in the KIN by Invergo *et al.* (2020) and 76.7% of the 5177 interactions in the KIN by Buljan *et al.* (2020). However, the KINference’s baseline KINs are also much larger, containing, respectively, 50 381 and 379 528 interactions. This leads to drops in OmniPath overlap from 13.3% to 2.5% and from 2.3% to 0.7% w.r.t. the KINs by Invergo *et al.* (2020) and Buljan *et al.* (2020), respectively. Overall, this shows that KINference’s baseline KINs recover the majority of the interactions in the KINs by Invergo *et al.* (2020) and Buljan *et al.* (2020), but also clearly indicates the need for the downstream filtering to improve the specificity.

**Figure 5.**
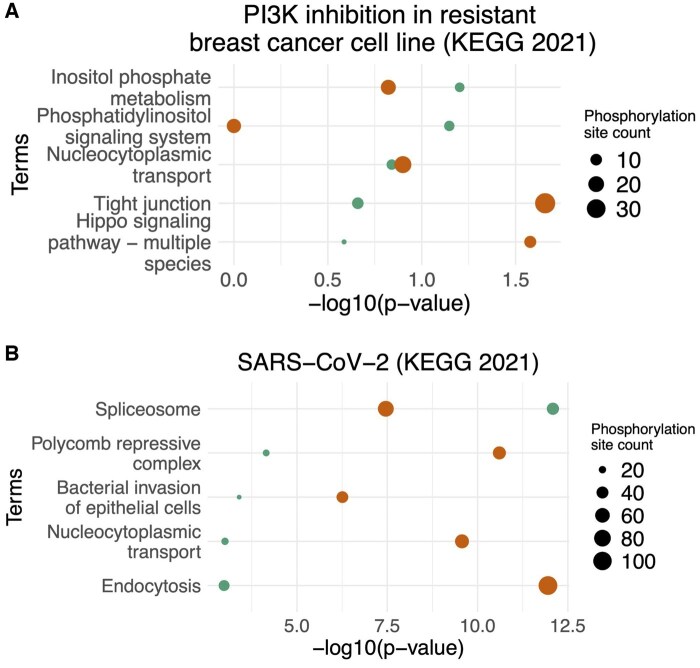
KEGG pathway enrichment of targeted proteins in DKINs (FS + DIFF + PCST, green dots) versus baseline KINs (orange dots).

GRNBoost2 returns a network of 360 538 edges when run on the data by Wilkes *et al.* (2015). 23.1% of these are also contained in the baseline KIN computed by KINference, which contains 263 404 interactions. Overlaps with OmniPath are similar for the two KINs (0.9% for GRNBoost2, 1.2% for KINference). Overall, this shows that the baseline KIN produces qualitatively comparable results to GRNBoost2, while being sample-size independent. However, GRNBoost2 also presents as a good alternative baseline KIN for KINference if there are enough samples. In view of this, we modularized the KINference R package such that every downstream filter can be used not only with KINference’s inbuilt baseline KIN but with any user-provided baseline KIN. The baseline KIN of KINference shows the lowest literature bias of all competitor methods (Fig. 5, available as supplementary data at *Bioinformatics* online).

### 3.3 KINference retrieves more known kinase–substrate interactions than random baselines

Figure 4 shows the results of our OmniPath-based evaluation of DKINs obtained with different filter combinations. When comparing KINference’s filters to random filtering of the baseline KINs (RF rows in Fig. 4A), we observe significantly larger overlaps with OmniPath for almost all tested filter combinations. The only exceptions are the DIFF filter and the PCST filter when used in isolation. The CORR filter cannot be used on the PI3K inhibition data because the data are provided as log2-fold changes. Joint application of node filters and edge filters yielded the best results for both test datasets. When compared to randomly rewired DKINs, all tested filter combinations yielded significantly larger overlaps with OmniPath on both datasets. This highlights that KINference indeed infers networks where information is encoded in the specific kinase–substrate links and which not merely contain well-annotated proteins as hubs—a problem previously identified for disease module mining on protein-protein interaction networks (Lazareva *et al.* 2021). Finally, KINference is able to significantly reduce the size of the baseline KINs, thereby facilitating hypothesis generation. For instance, the FS + DIFF + PCST filter combination reduces the sizes of the baseline KINs from $|E_{KS}^{\text{Wilkes}}|=71\;339$ and $|E_{KS}^{\text{Bouhaddou}}|=380\;835$ kinase–substrate interactions by >99% to $|E_{KS}^{⋆,\text{Wilkes}}|=472$ and $|E_{KS}^{⋆,\text{Bouhaddou}}|=2198$ edges (see Table 2, available as supplementary data at *Bioinformatics* online for DKIN sizes obtained with other filter combinations). The total number of nodes (kinases and other proteins) and the number of phosphorylation sites before and after applying any filters are provided in Table 3, available as supplementary data at *Bioinformatics* online.

### 3.4 KINference identifies functionally relevant kinase–substrate interactions

**Figure 6.**
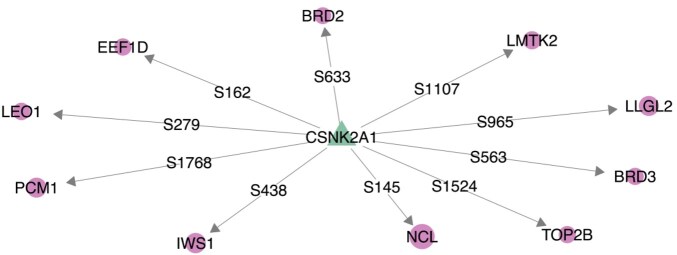
CSNK2A1—one of the most highly linked kinase in the DKIN inferred by KINference when run on the SARS-CoV-2 data by Bouhaddou *et al.* (2023)—together with its top 10 most differentially phosphorylated target sites (node sizes encode log2-fold changes, pink circular nodes represent target proteins, the edge labels specify the phorsphorylation sites).

Figure 5 shows the top enriched KEGG pathways for the target proteins of the KINs obtained when KINference is run with the combination of FS + DIFF + PCST filters (green dots) compared to the respective baseline KIN (orange dots) and their negative ${\;\text{log}\;}_{10}$-transformed adjusted *P*-values. The kinase source nodes were not included as they are already a highly specific set of nodes that leads to significantly enriched pathways (Fig. 6, available as supplementary data at *Bioinformatics* online). For the PI3K inhibition data, the first two terms “inositol phosphate metabolism” and “phosphatidylinositol signaling system” are more enriched in the DKIN than the same terms in the baseline KIN ($P_{\text{DKIN}}=0.0653$ versus $P_{\text{KIN}}=0.15$; $P_{\text{DKIN}}=0.07$ versus $P_{\text{KIN}}=1$). Furthermore, these terms are directly related to PI3K activity (Tu-Sekine and Kim 2022) and its signaling system (Nguyen *et al.* 2021). For the SARS-CoV-2 dataset, the top enriched term “Spliceosome” is more significant in the DKIN ($P_{\text{DKIN}}=1.3\times 10^{-12}$ versus $P_{\text{KIN}}=2.17\times 10^{-8}$) and is linked to SARS-CoV-2 infection (Banerjee *et al.* 2020). Overall, these results show that KINference indeed filters the network so that retained kinases and substrates are relevant in the context of user-provided data.

### 3.5 KINference suggests host pathways involved in SARS-CoV-2 infection

In addition to the quantitative analyses, we performed a case study on the SARS-CoV-2 data by Bouhaddou *et al.* (2023) to exemplify how KINference can be used for hypothesis generation. For this, we ran KINference with the FS + DIFF + PCST filter combination, obtaining a DKIN with around 2000 edges. With 49 regulated phosphorylation sites of 47 proteins (Fig. 6), casein kinase 2 alpha 1 (CSNK2A1)—a catalytic subunit of casein kinase 2 (CSNK2)—is the 8th most linked kinase behind SMG1 (73), MAPK14 (64), MAPK1 (61), BRAF (55), CSNK1G3 (54), CAMK2D (52) and CDK1 (51). All of these kinases can be linked to the SARS-CoV-2 infection through literature (Ma-Lauer *et al.* 2016, Su *et al.* 2021, Terracciano *et al.* 2021, Akbari *et al.* 2023, Konrat *et al.* 2023, Sui *et al.* 2023, Johnson *et al.* 2024). In order to find a starting point for hypothesis generation, we selected the top 10 linked kinases by out-degree and ranked the interactions based on the log2-fold change. The serine phosphorylation site NCL targeted by CSNK2A1 ranks the highest with a log2-fold change of 6.25. This result is very interesting in the light of studies by Yang *et al.* (2022) and Quezada Meza and Ruzzene (2023), where it was shown that CSNK2 inhibition can block SARS-CoV-2 replication. Since it has been shown that NCL interacts with viral proteins of SARS-CoV-2 and that inhibition of NCL decreases the viral reproduction (Merino *et al.* 2023), this suggests that CSNK2 may lead to a gain of function in NCL exploited by SARS-CoV-2. Although it has been shown that CSNK2 can phosphorylate NCL at serine sites (Xiao *et al.* 2014), no links between CSNK2 and phoshphorylation sites of NCL are annotated in OmniPath, highlighting KINference’s potential for *de novo* hypothesis generation.

## 4 Discussion and outlook

Here, we presented KINference, a tool for the data-specific inference of DKINs. While we have shown that KINference can retrieve high-quality context-specific DKINs, it is important to point out limitations of our method that can be improved in future work. Firstly, KINference relies on the PSSMs curated by Johnson *et al.* (2023) and Yaron-Barir *et al.* (2024) for inference of the baseline KINs. These PSSMs only contain 401 kinases in total and, thus, do not cover all of the over 500 human kinases. Furthermore, the experimental approach of Yaron-Barir *et al.* (2024) does not capture the effects of interpositional contacts. Interpositional contacts are two amino acids at two different positions surrounding the phosphorylation site of a targeted substrate of a kinase that jointly affect the binding probability of the kinase to this substrate. While Yaron-Barir *et al.* (2024) claim that interpositional contacts only marginally affect TYR kinases, contradicting findings have been reported in earlier studies (Miller *et al.* 2008). If emerging structural deep learning models such as AlphaFold3 (Abramson *et al.* 2024) will be able to reliably predict kinase–substrate interactions in the presence of interpositional effects, integration of such models into the baseline KIN inference step may allow future versions of KINference to model such effects between TYR kinases and to cover all human kinases.

Secondly, it could be interesting to replace the CORR filter by more sophisticated methods that also capture non-linear relationships between phosphorylation sites. Here, it may be possible to take inspiration from methods that infer GRNs from gene expression data. Early approaches to GRN inference also started out with correlation-based approaches when sample sizes were small (Langfelder and Horvath 2008). Afterwards, tree-based machine learning methods like GRNBoost2 (Moerman *et al.* 2019) became popular, which are still widely used today. Now, modern deep learning models like scGPT (Cui *et al.* 2024) are being developed to further improve GRN inference. All of these ideas could be transferred to the task of DKIN inference, once phosphoproteomics datasets with sufficiently large sample sizes become available.

## Supplementary Material

btaf349_Supplementary_Data

## Data Availability

All pre-processed data required to reproduce the results reported in this paper (PSSMs, background PSSM scores, phosphoproteomics data) are available on GitHub: https://github.com/bionetslab/KINference-Evaluation-Scripts. The original datasets are available in the supplementary information of the publications by Johnson *et al.* (2023), Wilkes *et al.* (2015), and Bouhaddou *et al.* (2023).

## Supplementary data

Conflict of interest: D.B.B. consults for BioVariance GmbH. The Krogan Laboratory has received research support from Vir Biotechnology, F. Hoffmann-La Roche, and Rezo Therapeutics. N.J.K. has a financially compensated consulting agreement with Maze Therapeutics. N.J.K. is the President and is on the Board of Directors of Rezo Therapeutics, and he is a shareholder in Tenaya Therapeutics, Maze Therapeutics, Rezo Therapeutics, GEn1E Lifesciences, and Interline Therapeutics.
