# Supplementary material for: Inference of differential kinase interaction networks with KINference
Source: Bioinformatics. 2025 Jun 20;41(7):btaf349. doi: 10.1093/bioinformatics/btaf349 (PMC12270260; doi:10.1093/bioinformatics/btaf349)
Supplement: btaf349_Supplementary_Data [file btaf349_supplementary_data.pdf]

Inference of differential kinase interaction networks with KINference

Supplementary information

1 Supplementary methods

1.1 Position specific scoring matrices (PSSM) scoring

For construction of the baseline KIN, KINference relies on data and methods provided and proposed by Johnson et al. [1] and Yaron-Barir et al. [2]. Johnson et al. performed positional scanning peptite analysis (PSPA) for $|K^{SER/THR}| = 303$ serine and threonine kinases. For this, Johnson et al. strategically fixed each of the nine positions (-5/+4) surrounding the central phosphorylation site. The central phosphorylation site contains equal amounts of phosphorylated serine and phosphorylated threonine. The surrounding positions were filled one by one with all possible 20 amino acids while the other positions contained approximately equimolar randomized mixtures of 17 amino acids excluding cysteine, serine and threonine. This generates a position-specific scoring matrix (PSSM) $P^k = (P^k_{a,i}) \in \mathbb{R}^{20\times 9}$ for each kinase $k \in K^{SER/THR}$, where higher values for a given position i and amino acid a lead to a higher likelihood of a kinase targeting a target sequence s.

The PSSMs can now be used to score a sequence s surrounding a phosphorylation site for each kinase $k \in K^{SER/THR}$ with:

$$r_{k,s} = \prod_{i=-5}^{4} P^k_{s_i,i}$$

However, this does not suffice to rank the likelihoods of all kinases targeting a sequence s because PSSM scores $r_{k,s}$ are not comparable between different kinases. To solve this, Johnson et al. computed background distributions $\{r_{k,s'} \mid s' \in S'\}$ for each kinase k utilizing a set of $|S'| = 82,735$ high-confidence phosphorylation sites detected using high-throughput MS by Ochoa et al. [3]. Now, the new sequence s can be scored and ranked in each of the background distributions of all kinases k as percentile scores $p_{k,s}$. These percentile scores can be ranked for all kinases $k \in K^{SER/THR}$, thus, giving a ranked list of the likelihoods for all kinases.

Yaron-Barir et al. executed the same experiment, but with phosphorylated tyrosine instead of phosphorylated serine and phosphorylated threonine at the central phosphorylation site and approximately equimolar randomized mixtures of 18 amino acids excluding cysteine and tyrosine. This yielded PSSMs for 93 tyrosine kinases $P^k \in \mathbb{R}^{20\times 9}$ for $k \in K^{TYR}$. Moreover, Yaron-Barir et al. used a set $|S'| = 5431$ of high-confidence tyrosine phosphorylation sites from Ochoa et al. [3]. Together, this is used in the exact same way as Johnson et al. to rank the likelihoods of the tyrosine kinases targeting tyrosine phosphorylation sites.

1.2 Comparison of KINference's baseline KIN against the two condition-agnostic KINs and the KIN inferred by GRNBoost2.

It is challenging to compare KINference to the condition-agnostic KIN inference methods of Invergo et al. [4] and the AP-MS-based KIN Buljan et al. [5], since KINference requires a phosphorylation site intensity measurements or fold changes as input, while Invergo et al. and Buljan et al. present two lists of kinase-protein interactions without phosphorylation site annotations. To evaluate the capabilities of the baseline KIN to recover these edges, we therefore constructed two mock datasets consisting of all possible phosphorylation sites of the high-confidence targeted proteins reported by Invergo et al. and Buljan et al., respectively. We used the same selection of high-confidence edges as Invergo et al. [4] (mean posterior probability greater or equal than 0.5) and Buljan et al. [5] (WD score ≥ 73.6, GFP-ratio ≥ 18.4). The resulting mock datasets consist of 37976 (Invergo et al.) and 255621 (Buljan et al.) phosphorylation sites. We did not use any of the data-specific filters because they require intensity measurements which are not given in these mock datasets. We considered an edge $e : k \to p$ of a kinase k to a protein p found by Invergo et al. [4] or Buljan et al. [5] recovered if at least one of the edges $e' : k \to p_s$ to a phosphorylation site s of p of the baseline KIN achieves a score greater than $\alpha = 0.9$ and ranks among the top $n = 15$ interactions. Furthermore, we only considered theoretically recoverable edges for the comparison by removing all interactions from the KINs by Invergo et al., Buljan et al., and the baseline KIN, where the source kinase is not among the kinase motifs of Johnson et al. [1] and Yaron-Barir et al. [2].

On the data of Bouhaddou et al. [7], we additionally compare KINference's baseline KIN against the state-of-the-art network reconstruction tool GRNBoost2, originally designed to infer gene regulatory networks (GRNs) from transcriptomics data. We restricted the regulators to the phosphorylation sites s_k of all kinases k and ran GRNBoost2 on the samples from both conditions individually. We added an edge $e : k \to s$ from a kinase k to a phosphorylation site s if GRNBoost2 returns an edge $e' : s_k \to s$ in either of the two runs, where s_k is a phosphorylation site of k. This ensures that the KIN inferred by GRNBoost only contains physiologically relevant edges and is in the same format as the baseline KIN inferred by KINference.

2 Supplementary results

2.1 Supplementary figures

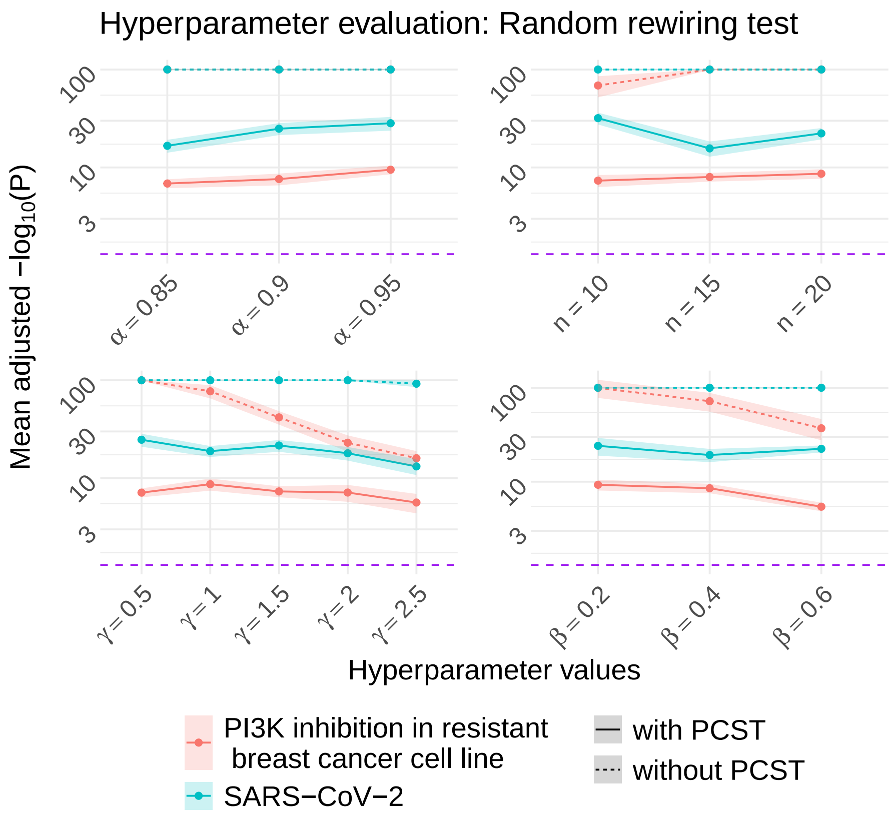

Supplementary Figure 1: Hyperparameter evaluation of the randomized rewiring test of the hyperparameters α, n, β and γ for the two test datasets utilizing DIFF + FS (dashed) and PCST + DIFF + FS (solid). Mean adjusted $-log_{10}$-transformed P-values and 5% confidence intervals are shown.

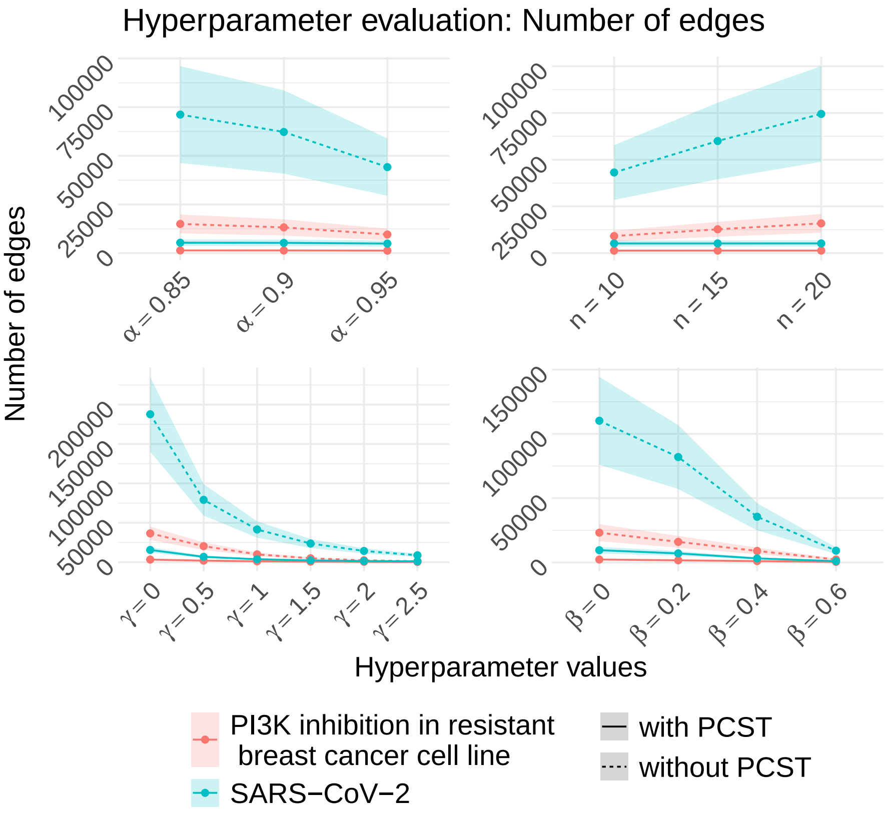

Supplementary Figure 2: Hyperparameter evaluation of the randomized filter test of the hyperparameters α, n, β and γ for the two test datasets utilizing DIFF + FS (dashed) and PCST + DIFF + FS (solid). Number of edges and 5% confidence intervals are shown.

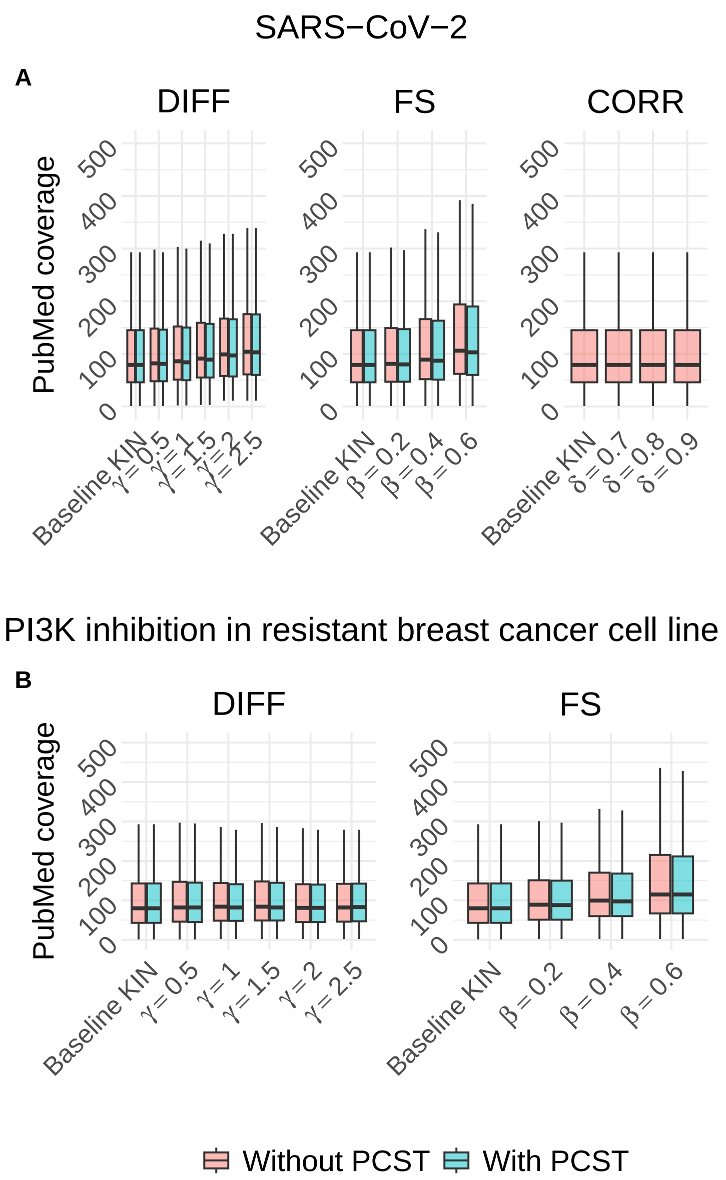

Supplementary Figure 3: Hyperparameter evaluation of the literature bias of the hyperparameters β , γ, and δ for the SARS-CoV-2 test dataset (A) and β and γ for the PI3K inhibition in resistant breast cancer cell line test dataset (B). The boxplots show the number of time each target protein is mentioned in PubMed. See Supplementary Table 3 for the results of corresponding correlation test that asses the association strength between the hyperparameters controlling the filters and the PubMed coverages.

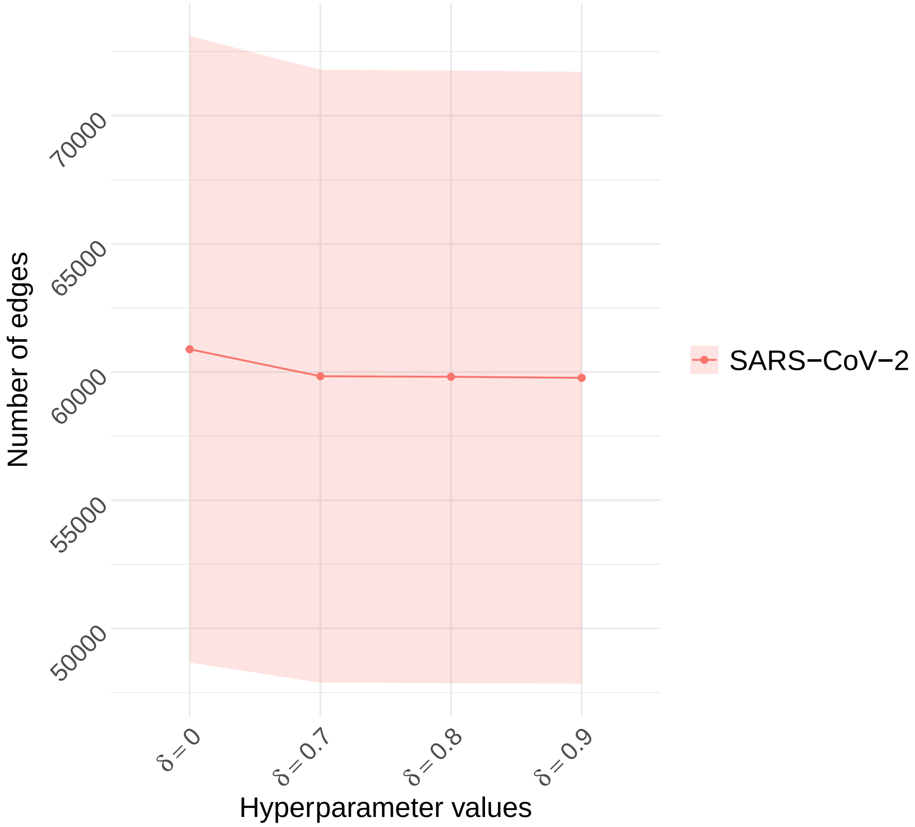

Supplementary Figure 4: Hyperparameter evaluation of the effect size for different values of δ.

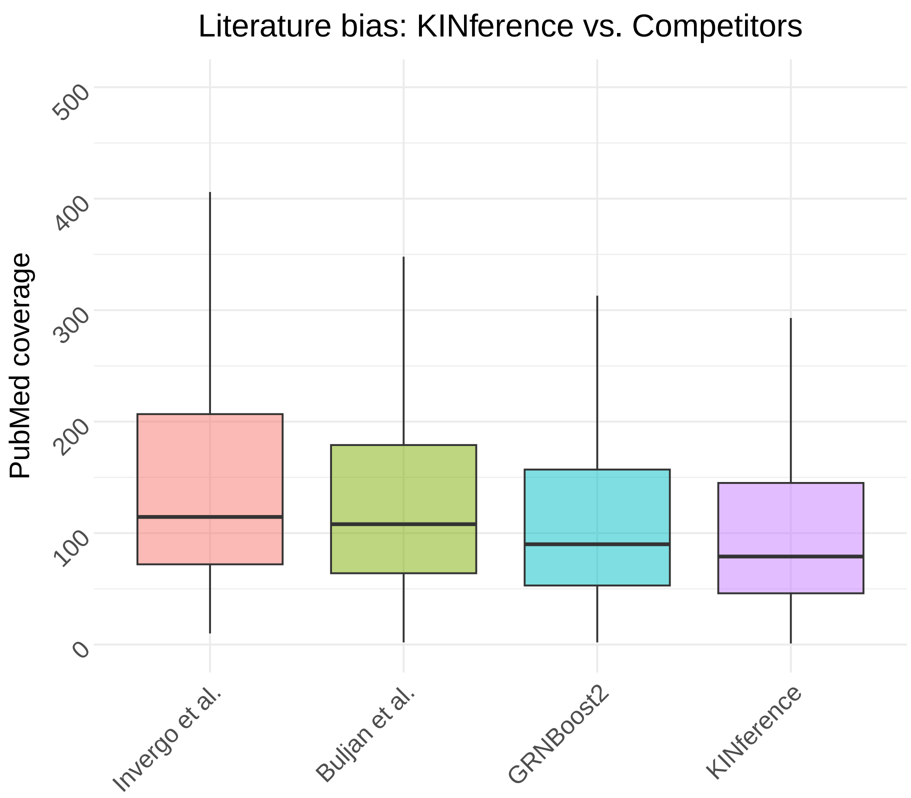

Supplementary Figure 5: Literature bias evaluation of KINference vs. competitor methods for baseline KIN generation. The boxplots show the numbers of PubMed IDs which are indexed to target proteins contained in the different networks.

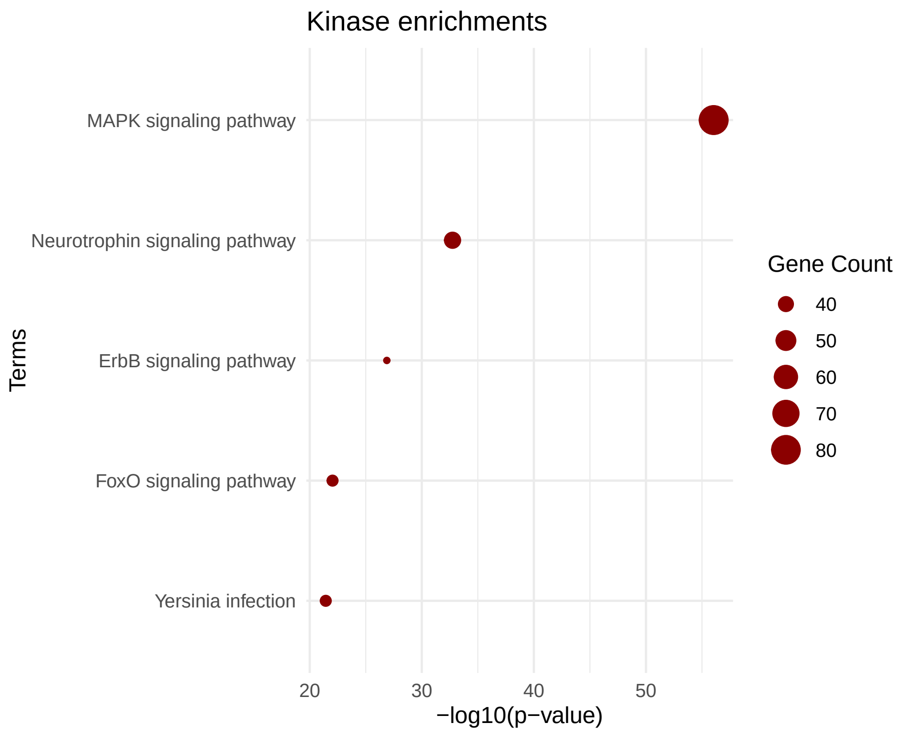

Supplementary Figure 6: Enriched KEGG pathways when running g:Profiler with all kinases K contained in the baseline KIN as input.

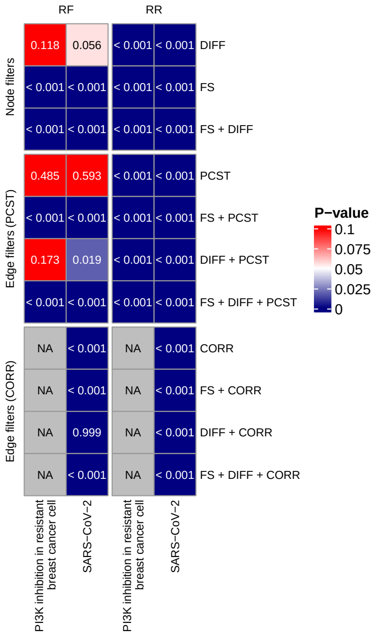

Supplementary Figure 7: P-values (one-sided Z-test) obtained when comparing OmniPath overlaps achieved with KINference and all possible combinations of node and edge filters against overlaps achieved via random filtering (RF) of the baseline KIN and randomized rewiring (RR) of the KINs computed by KINference.

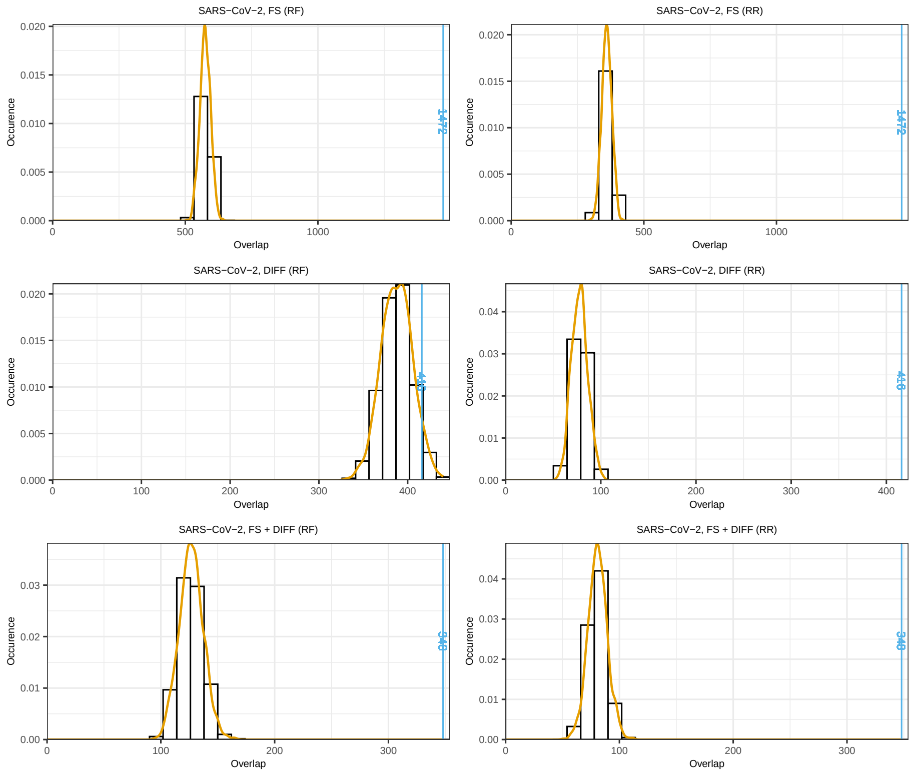

Supplementary Figure 8: Distributions of OmniPath overlaps underlying the P-values for all combinations of the the FS and DIFF node filters on the SARS-CoV-2 test dataset. Histograms and density plots visualize the overlap distributions obtained with the randomized baselines; the blue lines show the overlaps of the KINs computed with KINference.

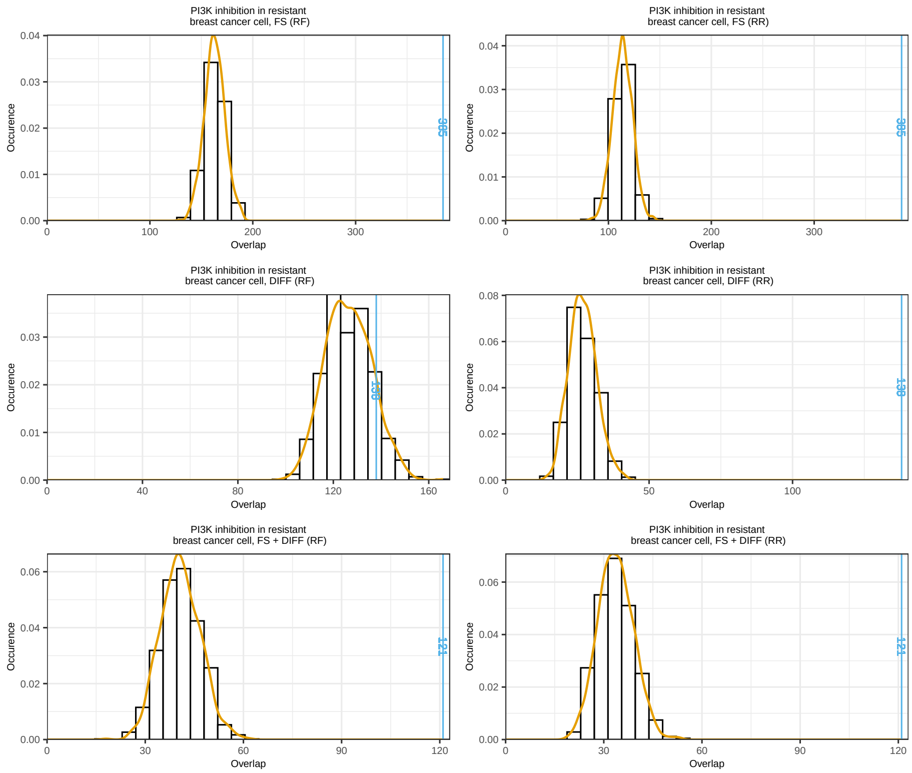

Supplementary Figure 9: Distributions of OmniPath overlaps underlying the P-values for all combinations of the the FS and DIFF node filters on the PI3K inhibition in resistant breast cancer cell line test dataset. Histograms and density plots visualize the overlap distributions obtained with the randomized baselines; the blue lines show the overlaps of the KINs computed with KINference.

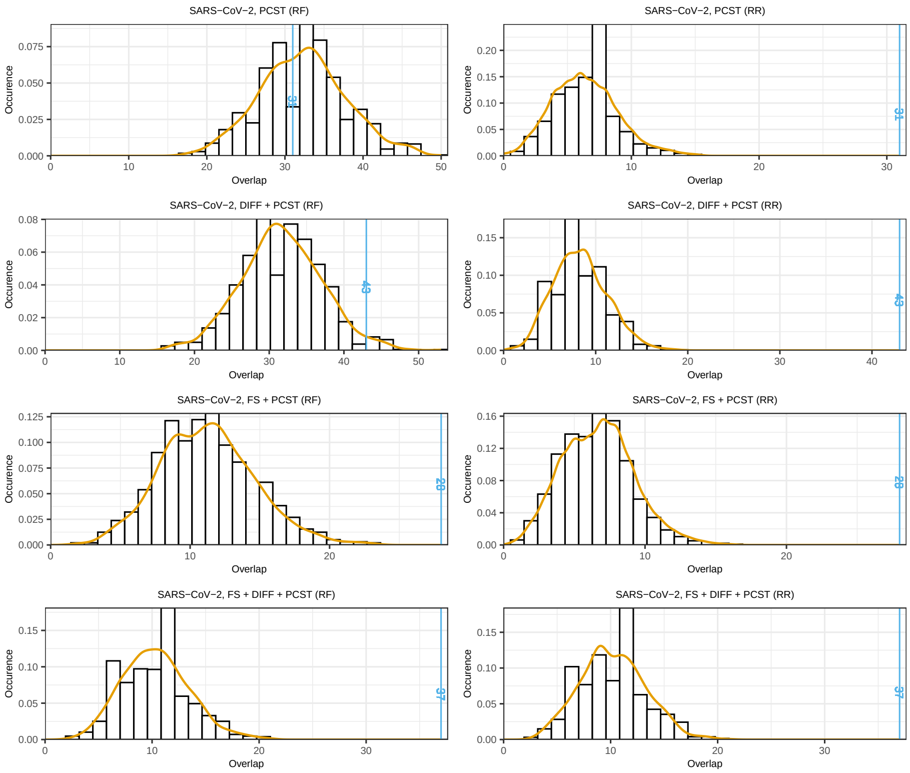

Supplementary Figure 10: Distributions of OmniPath overlaps underlying the P-values for all combinations of the the FS, DIFF and PCST filters on the SARS-CoV-2 test dataset. Histograms and density plots visualize the overlap distributions obtained with the randomized baselines; the blue lines show the overlaps of the KINs computed with KINference.

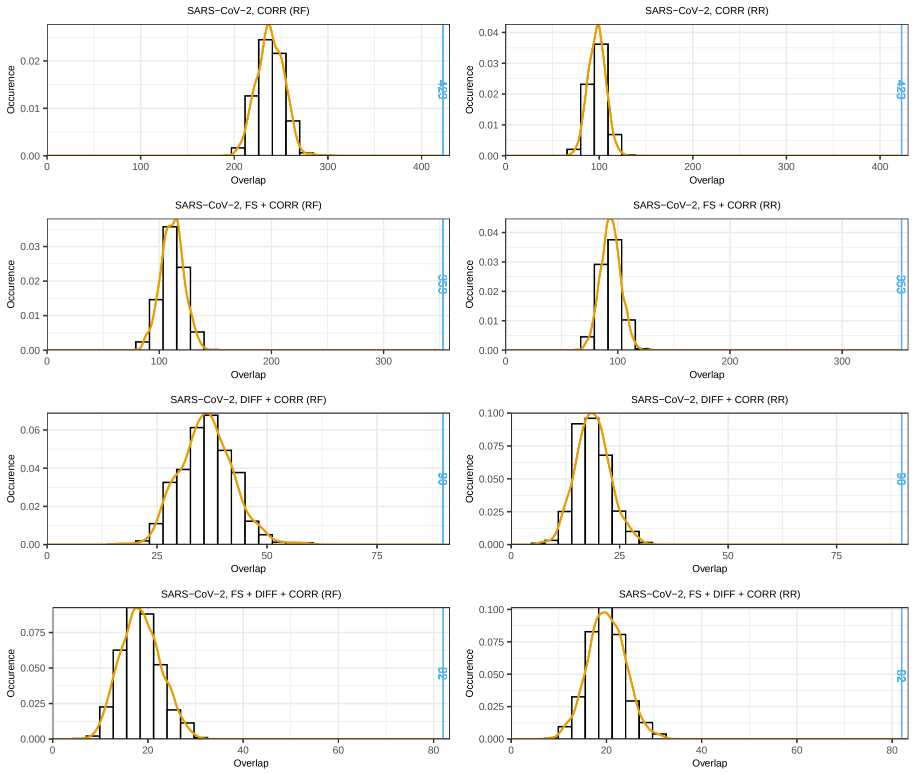

Supplementary Figure 11: Distributions of OmniPath overlaps underlying the P-values for all combinations of the the FS, DIFF and CORR filters on the SARS-CoV-2 test dataset. Histograms and density plots visualize the overlap distributions obtained with the randomized baselines; the blue lines show the overlaps of the KINs computed with KINference.

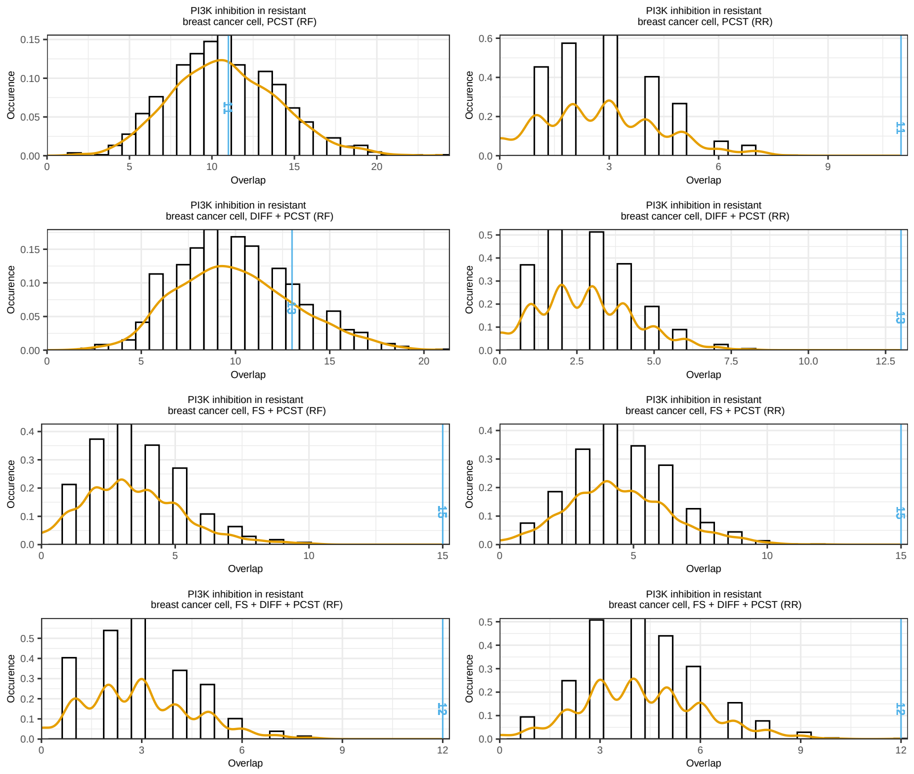

Supplementary Figure 12: Distributions of OmniPath overlaps underlying the P-values for all combinations of the the FS, DIFF and PCST filters on the PI3K inhibition in resistant breast cancer cell line test dataset. Histograms and density plots visualize the overlap distributions obtained with the randomized baselines; the blue lines show the overlaps of the KINs computed with KINference.

2.2 Supplementary tables

Supplementary Table 1: Results of two-sided Pearson correlation tests to assess the association between hyperparameters controlling KINference's filters and the PubMed coverages of target proteins in the resulting DKINs (see Supplementary Figure 10 for the corresponding boxplots). Correlation tests were carried our using the R function `cor.test()`. The P-values are adjusted for multiple testing via Benjamini-Hochberg to account for the number of tested filter combinations.

	PI3K inhibition in resistant breast cancer cell line (Pearson's correlation coefficient)	PI3K inhibition in resistant breast cancer cell line (adjusted P−value)	SARS−CoV−2 (Pearson's correlation coefficient)	SARS−CoV−2 (adjusted P−value)
FS	0.0904	1.4e−06	0.0604	1.27e−07
DIFF	0.0393	0.0754	0.0264	0.0517
CORR	NA	NA	−5.51e−16	1
FS + PCST	0.0973	3.45e−07	0.0607	2.57e−07
DIFF + PCST	0.0412	0.0632	0.0391	0.00104

Supplementary Table 2: Number of interactions that are left after applying each possible filter combination. The second and fourth column show the reduction in percent compared to the baseline KIN.

	PI3K inhibition in resistant breast cancer cell line	Reduction (%)	SARS−CoV−2	Reduction (%)
Baseline KIN	71339	0%	380835	0%
FS	25809	63.82%	121521	68.09%
DIFF	19955	72.03%	82099	78.44%
FS + DIFF	6460	90.94%	26761	92.97%
CORR	NA	NA	50549	86.73%
FS + CORR	NA	NA	23579	93.81%
DIFF + CORR	NA	NA	7686	97.98%
FS + DIFF + CORR	NA	NA	3927	98.97%
PCST	1678	97.65%	6816	98.21%
FS + PCST	551	99.23%	2347	99.38%
DIFF + PCST	1581	97.78%	6696	98.24%
FS + DIFF + PCST	472	99.34%	2198	99.42%

Supplementary Table 3: Total number of nodes (kinases / non-kinases) and total number of phosphorylation sites (phosphorylation sites of kinases / phosphorylation sites of non-kinases) that are left after applying each possible filter combination.

	PI3K inhibition in resistant breast cancer cell line (no. proteins)	PI3K inhibition in resistant breast cancer cell line (no. phosphorylation sites)	SARS−CoV−2 (no. proteins)	SARS−CoV−2 (no. phosphorylation sites)
Baseline KIN	2849 (393 / 2456)	5823 (198 / 5625)	4735 (393 / 4342)	31406 (1370 / 30036)
FS	1408 (392 / 1016)	2026 (116 / 1910)	3469 (393 / 3076)	9903 (591 / 9312)
DIFF	1416 (385 / 1031)	1598 (43 / 1555)	2778 (393 / 2385)	6759 (234 / 6525)
FS + DIFF	733 (358 / 375)	509 (24 / 485)	1665 (393 / 1272)	2221 (108 / 2113)
CORR	NA	NA	2904 (135 / 2769)	10096 (442 / 9654)
FS + CORR	NA	NA	2167 (135 / 2032)	4736 (260 / 4476)
DIFF + CORR	NA	NA	1052 (131 / 921)	1697 (55 / 1642)
FS + DIFF + CORR	NA	NA	712 (130 / 582)	871 (35 / 836)
PCST	1161 (120 / 1041)	1564 (5 / 1559)	2621 (226 / 2395)	6595 (8 / 6587)
FS + PCST	444 (70 / 374)	484 (3 / 481)	1477 (187 / 1290)	2168 (11 / 2157)
DIFF + PCST	1120 (131 / 989)	1460 (9 / 1451)	2585 (205 / 2380)	6503 (15 / 6488)
FS + DIFF + PCST	391 (71 / 320)	412 (10 / 402)	1386 (141 / 1245)	2069 (14 / 2055)

References

[1] Johnson et al. (2023) An atlas of substrate specificities for the human serine/threonine kinome. 10.1038/s41586-022-05575-3

[2] Yaron-Barir et al. (2024) The intrinsic substrate specificity of the human tyrosine kinome. 10.1038/s41586-024-07407-y

[3] Ochoa et al. (2020) The functional landscape of the human phosphoproteome. 10.1038/s41587-019-0344-3

[4] Invergo et al. (2020) Prediction of signed protein kinase regulatory circuits. *Cell systems*, 10(5):384–396, 2020.

[5] Buljan et al. (2020) Kinase Interaction Network Expands Functional and Disease Roles of Human Kinases. 10.1016/j.molcel.2020.07.001

[6] Cui et al. (2024) scGPT: toward building a foundation model for single-cell multi-omics using generative AI. 10.1038/s41592-024-02201-0

[7] Bouhaddou et al. (2023) SARS-CoV-2 variants evolve convergent strategies to remodel the host response. 10.1016/j.cell.2023.08.026

[8] Wilkes et al. (2015) Empirical inference of circuitry and plasticity in a kinase signaling network. 10.1073/pnas.1423344112
